# Overlap of psoriasiform and primary syphilis: an atypical manifestation of secondary syphilis (a case report)

**DOI:** 10.11604/pamj.2022.42.229.36116

**Published:** 2022-07-26

**Authors:** Rizki Amelia Noviyanthi, Ivan Kurniadi, Muji Iswanty, Safruddin Amin, Anni Adriani, Muhlis

**Affiliations:** 1 Department of Dermatology and Venereology, Faculty of Medicine, Universitas Hasanuddin, Makassar, South Sulawesi, Indonesia

**Keywords:** Overlap, HIV, psoriasiform, syphilis, case report

## Abstract

Syphilis is a sexually transmitted infection caused by the bacterium Treponema pallidum (T. pallidum) with an increasing incidence in recent years. Secondary syphilis is called 'the great imitator' due to its various clinical presentations. Psoriasiform syphilis is an atypical presentation of secondary syphilis. The coinfection of syphilis with HIV has been linked to more severe clinical presentations increased risk of neurosyphilis, decreased CD4+ count, and an interesting phenomenon of overlapping primary and secondary syphilis. A 35-year-old male presented with generalized thick, scaly erythematous plaques, including the soles of the palms and feet, diffuse alopecia on the scalp and eyebrows, and multiple painless ulcers on the penis. The venereal disease research laboratory and Treponema pallidum hemagglutination assay examination showed positive results and the patient was treated with an intramuscular injection of 2.4 million units of Benzathine penicillin G. At the seventh-day follow-up, the patient showed significant clinical improvement marked by plaque thinning and reduced erythema. This case emphasizes that secondary syphilis may present with varied clinical presentations which can be further affected with HIV coinfection. Detailed history taking, physical examination, and a high level of suspicion are crucial in recognizing and establishing the right diagnosis.

## Introduction

Syphilis is a chronic sexually transmitted infection (STI) caused by the bacterium *Treponema pallidum*. The incidence of syphilis has tended to increase over the past few years. During 2013-2014, primary and secondary syphilis rates increased by 14.4% in men and 22.7% in women [1]. In addition, the incidence of syphilis nearly doubled during 2013-2017 [2]. Based on epidemiological data in 2016, the incidence of syphilis in Indonesia amounted to 7,055 cases [3]. This disease is divided into primary, secondary, and tertiary stages. Of these three phases, secondary syphilis is referred to as 'the great imitator' because of its highly variable clinical presentation [4]. The most common symptom is termed roseola syphilitica, which is a generalized maculopapular rash that can be accompanied by constitutional symptoms such as fever, malaise, and myalgia [1]. Other rare clinical presentations such as Biette's collarette, lichenoid, hyperkeratotic, psoriasiform, even overlap of primary and secondary lesions may occur [1]. People infected with syphilis should also be checked for HIV infection because both diseases are contracted through sexual intercourse. In general, the clinical presentation and therapy of syphilis in people with HIV are the same [5]. However, co-infection of syphilis with HIV has been associated with heavier clinical presentation, overlap of primary and secondary syphilis, increased risk of neurosyphilis, and lower CD4+ count [6]. This case report describes an overlapping of psoriasiform syphilis and primary syphilis lesions in an HIV-positive patient.

## Patient and observation

**Patient information**: a 35-year-old man presented with non-pruritic generalized scaly red patches in the last one month. Initially the lesions appeared on the face which then extended to other parts of the body, including the palms and soles. The patient also complained of hair loss since the appearance of the patches. Past and family history of similar lesions were absent. A history of painless genital ulcer was denied. The patient was diagnosed with HIV two months prior to admission and had been on antiretroviral therapy (ART). However, the ART was temporarily discontinued due to suspected drug allergy. The patient admitted to having the last sexual encounter three months prior to admission with his wife. He also reported sexual contact with prostitutes without protection.

**Figure 1:**
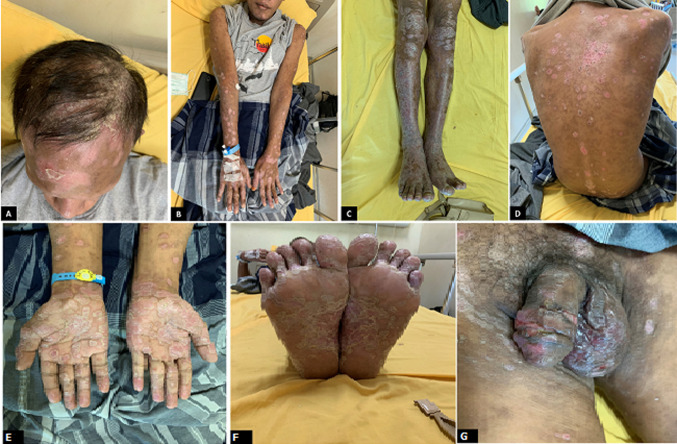
lesions on the first day; (A) erythematous plaques and alopecia on the scalp; (B) erythematous plaques with thick scales on the superior extremities; (C) inferior extremities; (D) trunk; (E) scaly erythematous plaques on both palms; (F) soles; (G) non-painful multiple ulcers on the penis

**Clinical findings**: on physical examination, the patient was in a good general state with normal vital signs. Dermatological examinations showed generalized squamous annular erythematous plaques and erythematous macules (Figure 1). Diffuse nonscarring alopecia was observed on the scalp. Multiple ulcers and erythematous plaques were observed on the penis.

**Figure 2:**
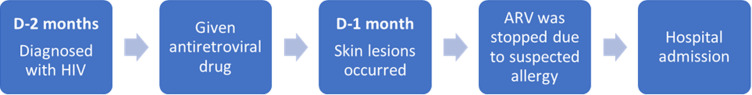
history of disease

**Timeline of current episode**: history of disease is described as a timeline (Figure 2).

**Diagnostic assessment**: blood examination indicated anemia, hypoalbuminemia, and hypoglobulinemia. Syphilis serologic examination results showed positive VDRL with titer 1: 128 and TPHA.

**Diagnosis**: based on the results of anamnesis and physical examination, the patient was diagnosed with psoriasiform secondary syphilis.

**Therapeutic interventions**: the patient was given 2.4 million units of intramuscular benzathine G penicillin injection on the right and left gluteus. The ART was planned to be resumed.

**Figure 3:**
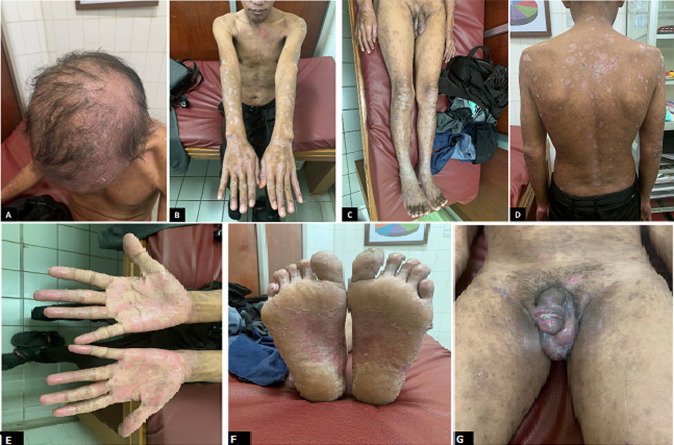
lesions on the seventh day of erythematous plaque; (A) the scalp; (B) superior extremities; (C) inferior extremities; (D) trunk had thinned, thick scaly erythematous plaques; (E) palms; (F) soles had also thinned; non-painful multiple ulcers; (G) the penis appeared shallower and almost resolved

**Follow-up and outcome of interventions**: the next day the patient experienced a high fever without the appearance of erythematous macules which subsided following paracetamol administration. On the seventh day, the skin lesions showed significant improvement (Figure 3). The erythematous plaques had thinned and decreased in number; ulcers on the penis looked shallower. A follow-up VDRL titer examination was planned in three months. In addition, the patient's wife was also recommended to do a syphilis and HIV screening examination.

**Patient perspective**: the patient felt so relieved that the extensive rash and alopecia could eventually be resolved. Aside from the painful Benzathine Penicillin injection, he did not have any complaints about the whole treatment regimen.

**Informed consent**: the patient had given consent for publication.

## Discussion

Cutaneous manifestation is the most common clinical picture of secondary syphilis. The clinical picture varies greatly, hence the term “great imitator” [6]. The most frequent clinical presentation is macular or maculopapular erythematous rash that arises on the torso and extremities [1]. In rare cases, when the scale is thicker, the lesion may resemble psoriasis (psoriasiform) [4], as found in this case. The presence of lesions on the palms and soles is a strong indication of syphilis [5,6]. In addition to the trunk and extremities, the patient also complained of hair loss of the head and eyebrows. Alopecia is a phenomenon that can occur in syphilis with a typical moth-eaten appearance. However, diffuse-type alopecia, although less frequently, can also occur. In addition to the head, alopecia can also arise on the eyebrows, especially the lateral third [1,5]. The pathogenesis of syphilitic alopecia has not been clearly understood [7], but it is suspected that there is a specific immunological reaction in the *T. pallidum* antigen, which is indicated by the discovery of *T. pallidum* DNA in the hair follicles [8]. In addition, immunohistochemical examination shows spirochete on the hair follicles which indicates the direct role of *T. pallidum* inducing hair loss [8-10].

Multiple painless erosions were found on the genitalia of the patient. Non-painful genital ulcers (chancre) are typically found in primary syphilis; however, in co-infection with HIV, as in this case, chancre that commonly occur in primary syphilis may appear along with secondary syphilis symptoms [6,11]. In addition, syphilitic chancres which are generally only solitary and indurated can be multiple in number, be wider in size, and develop in a more aggressive fashion [6,12]. The patient responded well to intramuscular injection of 2.4 million-unit Benzathine G penicillin. According to the National Guidelines for 2015 and CDC in 2015, Benzathine G penicillin is the first-line therapy in primary and secondary syphilis. The effectiveness of this drug in syphilis therapy has been time-tested and has become standard therapy on almost all guidelines. In addition, Benzathine G penicillin has a long half-life corresponding to the long dividing time of *T. pallidum* [1].

A few hours after therapy symptoms of the Jarisch-Herxheimer reaction were observed. This reaction was signified by headaches, muscle pain, and other symptoms that can arise in the first 24 hours post-therapy [13]. The pathophysiology of the Jarisch-Herxheimer reaction is still uncertain but is thought to have occurred as a result of cytokine release mediated by lipoproteins from dead *T. pallidum* [1]. The incidence of the Jarisch-Herxheimer reaction was found to be higher in people with HIV [14]. Treatments include symptomatic approach which usually includes antipyretics and/or nonsteroidal anti-inflammatory drugs (NSAID) [1]. In this case, the patient responded well to paracetamol and experienced prompt improvement.

## Conclusion

Secondary syphilis is a great imitator because it can present with varying clinical presentations which can be further complicated by HIV co-infection. A thorough history taking and physical examination accompanied by a high level of suspicion are crucial in recognizing and enforcing the right diagnosis.
